# Using qualitative evaluation components to help understand context: case study of a family planning intervention with female community health volunteers (FCHVs) in Nepal

**DOI:** 10.1186/s12913-020-05466-1

**Published:** 2020-07-23

**Authors:** Cicely Marston, Abriti Arjyal, Smriti Maskey, Shophika Regmi, Sushil Baral

**Affiliations:** 1 London School of Hygiene & Tropical Medicine, 15-17 Tavistock Place, London, WC1H 9SH UK; 2 HERD International, Thapathali, Kathmandu, Nepal

**Keywords:** Evaluation, Contraception, Women’s health, Community outreach

## Abstract

**Background:**

Evaluations of health interventions are increasingly concerned with measuring or accounting for ‘context’. How to do this is still subject to debate and testing, and is particularly important in the case of family planning where outcomes will inevitably be influenced by contextual factors as well as any intervention effects. We conducted an evaluation of an intervention where female community health volunteers (FCHVs) in Nepal were trained to provide better interpersonal communication on family planning. We included a context-orientated qualitative component to the evaluation. Here, we discuss the evaluation findings, specifically focusing on what was added by attending to the context. We explore and illustrate important dimensions of context that may also be relevant in future evaluation work.

**Methods:**

The evaluation used a mixed methods approach, with a qualitative component which included in-depth interviews with women of reproductive age, FCHVs, and family planning service providers. We conducted iterative, thematic analysis.

**Results:**

The life-history fertility and contraception narratives generated from the in-depth interviews contextualised the intervention, yielding nuanced data on contraceptive choices, needs, and areas for future action. For instance, it highlighted how women generally knew about effective contraceptive methods and were willing to use them: information was not a major barrier. Barriers instead included reports of providers refusing service when women were not in the fifth day of their menstrual cycle when this was unnecessary. Privacy and secrecy were important to some women, and risked being undermined by information sharing between FCHVs and health services. The qualitative component also revealed unanticipated positive effects of our own evaluation strategies: using referral slips seemed to make it easier for women to access contraception.

**Conclusions:**

Life history narratives collected via in-depth interviews helped us understand pathways from intervention to effect from the user point of view without narrowly focusing only on the intervention, highlighting possible areas for action that would otherwise have been missed. By attending to context in a nuanced way in evaluations, we can build a body of evidence that not only informs future interventions within that context, but also builds better knowledge of contextual factors likely to be important elsewhere.

## Background

Evaluations of health interventions are increasingly concerned with measuring or accounting for ‘context’ [1]. How to do this is still subject to debate and testing, and is particularly important in the case of family planning where outcomes will inevitably be influenced by contextual factors as well as any intervention effects. Evaluation of family planning programmes often focus on measuring ‘impact’ of interventions on contraceptive uptake [2]. Yet the interventions intended to improve contraceptive uptake may be very distal from the uptake itself and the causal pathways between the intervention and the ultimate goal of improving uptake are not always well specified. Understanding how an intervention might improve family planning, or understanding why it has not worked as planned requires attention to context. Here we discuss how we incorporated an in-depth qualitative component to a family planning evaluation to attempt to account for women’s lives and experiences connected with fertility, contraception and abortion. The aim was to provide a person-centred view of the context of the intervention to understand how the intervention might shape and be shaped by women’s lives and experiences.

Our evaluation concerned the effects of an interpersonal communication (IPC) training intervention for female community health volunteers (known locally by the initialism FCHV) on uptake of contraception. We faced considerable challenges from the outset. First, we judged it unlikely that the intervention would have a measurable impact on contraceptive uptake or fertility at the population level over the short timeframe of the programme evaluation (just over 6 months). Second, even if there were a measurable effect, the evaluation budget was insufficient to measure population-level changes. Third, the implementation agency wished to conduct the intervention in the highest need areas (areas selected for low family planning uptake, presence of marginalised groups, access to services to meet any increased demand) and not randomly across the district, making a trial design challenging to implement. Fourth, although information about family planning options is important, lack of information is not the only reason women do not use reliable methods to prevent pregnancy [3, 4] and so focusing narrowly on the role of information would likely miss key features of contraceptive use. We decided to include a substantial qualitative component in the evaluation, partly to mitigate the challenges we faced and conscious of the limitations of other options, but also experimentally to try to understand both the setting and the programme better in the absence of so-called ‘gold-standard’ (which at the time had come to mean only randomised controlled trial) evaluation possibilities. Here we focus particularly on the contribution of the qualitative component, highlighting how it generated unexpected insights and contextual detail. Our aim in this paper is not simply to illustrate contextual features of contraceptive use in Nepal, but also to show how qualitative approaches to evaluation have the potential to improve understanding of interventions and their context.

### Nepal family planning and female community health volunteers

The evaluation we examine here was conducted in Myagdi, a hill area of Nepal. It involved training and supporting FCHVs to provide better information about contraception to people in their local communities. The network of FCHVs was set up in the 1980s to help reach the large rural population, particularly women and children [5]. FCHVs do not receive any regular pay for their work. They are tasked with conducting health promotion activities, particularly for family planning, child health, and safe motherhood [6]. New FCHVs receive 18 days of basic primary health care training [7]. On completion, they are given a medicine kit box with a few essential drugs and supplies, manuals, flip charts, a ward register, information materials, FCHV bag, a sign board and an identity card. They distribute condoms and resupply current users with oral contraceptive pills (OCPs) supplied by the government. More than 50% of all OCPs are distributed by FCHVs [8]. FCHVs average 41.3 years old; 67% have any level of schooling [9]. Over 59% of FCHVs recently reported over 10 years of service [9].

As part of their regular work, FCHVs are asked to promote six methods of contraception (condoms, OCPs, injectables, implants, intrauterine contraceptive devices (IUCDs), permanent methods); there is also a brief mention of emergency contraception in the FCHV manual [6]. Almost all (97%) FCHVs in a national survey said they had provided at least some family planning services in the preceding 3 months [9]. One of the FCHV activities is conducting so-called ‘health mothers group’ meetings. This involves convening a group of local women of reproductive age to provide them with health information and advice, which can include discussions of family planning. The FCHV National Survey identified several barriers to FCHV work, including the uneven supply of commodities (e.g. condoms and OCPs); lack of good supervision of FCHV work; lack of understanding of FCHV roles on the part of FCHVs themselves and other stakeholders [9]. Family planning promotion is a core FCHV task. Yet many were trained years ago, and refresher training is inconsistent.

According to Nepal Government policy, all health facilities should provide five temporary methods of contraception: condoms, OCPs, injectables, IUCDs, and implants [10]. Permanent methods are provided in hospitals or via ‘sterilisation camps’ where providers visit locations on specific dates to offer these methods [11]. Abortion is legally permitted in Nepal up to 12 weeks of gestation on request, up to 18 weeks in case of rape or incest, and any time if the pregnancy poses a danger to the woman’s life or physical or mental health, or if there is a foetal abnormality [12]. In practice, policy goals may not always be met because of stockouts or lack of trained providers [13]. At the time of the project, abortion services had to be provided via government-accredited health facilities and service providers [14]. Myagdi district contained a primary health care centre (PHCC) in Darwang and a district-level hospital in Beni, with health posts elsewhere. There were 13 health facilities (including the hospital and PHCC) authorised to provide medication abortion in Myagdi; the hospital also provided surgical abortion services. The PHCC should have provided surgical abortion but the required infrastructure was absent so it did not. Technical support to provide medication abortion to the 13 locations was being provided by IPAS, a non-profit organisation. Stockouts and other difficulties can be especially acute in the more remote hill and mountain areas where it can be difficult to retain trained staff and where logistics are challenging.

In this context, a pilot interpersonal communication training intervention was implemented by the Health Communication Capacity Collaborative (HC3) in collaboration with the District Health Office, Myagdi [15]. The aim was to improve FCHV knowledge and interpersonal communication on family planning and promote family planning to their communities, and by doing so, improve uptake of modern contraceptive methods. Our task was to evaluate the impact of this pilot. The pilot was carried out under the previous government structure in which districts were divided into Village Development Committees (VDCs) subdivided into wards. Myagdi contained 31 VDCs and one municipality (Beni, formed of what had been 6 VDCs). At the time of the study, there was at least one FCHV per 1000 population [16], with at least one FCHV per ward. Ward sizes in Myagdi ranged from 46 to 5400 [17]. In each of the VDCs, FCHVs received training in interpersonal communication skills to promote family planning. Training was two days long and covered basic information about contraceptive methods and information on communication skills. Fifteen of the VDCs additionally received an ‘intensive’ version of the intervention which provided supportive supervision from project staff of the implementing agency to FCHVs to help them carry out their tasks including conducting ‘health mothers groups’, drawing up social maps to identify eligible women, helping them communicate appropriately about family planning, and distribute educational materials, as well as helping them refer and follow up eligible women. More details of the pilot and the evaluation can be found in the final evaluation report [15].

## Methods

We conducted a qualitative study embedded within an evaluation of the FCHV IPC training intervention in Myagdi district, Nepal. The massive earthquake of April 2015 overshadowed the year, and disrupted intervention activities as efforts were diverted to disaster relief. Myagdi district was not one of the districts most damaged by the earthquake, although it did suffer disruption. Shortly afterwards in May 2015, a landslide blocked a river, causing severe flooding and further disruption.

### Data collection

We had prepared for data collection via planning meetings with healthcare staff and similar, prior to the earthquake and landslide. We collected qualitative data afterwards, from August 2015 to January 2016. For practical (time and resource) reasons unconnected with the earthquake, we focused on three VDCs for the qualitative enquiry: Darwang, Singa, and Rakhu Bhagwati. We conducted 21 one-to-one, in-depth interviews with women of reproductive age (15–49 years) from the wards of these focal VDCs. FCHVs helped us find five women they had already referred for family planning services and we recruited other women by using local contacts and snowball sampling. We sampled for diversity in parity, age, residence and ethnicity (see Table 1). Excerpts from these interviews are labelled ‘IDI’ (in-depth interview) in the text. The only inclusion criterion was that they were women of reproductive age – they did not have to have used contraceptive methods.

**Table 1:** Characteristics of interviewees: women of reproductive age

Characteristic	N
**Age**	
17–20 years	2
Over 20 years	19
**Highest education level**	
No formal education	4
Primary level	3
Lower secondary level	8
Secondary (SLC completed)	3
Intermediate level	3
**Number of children**	
0	2
1	5
2	8
3	4
4	2
**Ever used contraceptive method**	
Yes	15
No	6
**Caste/ethnicity**	
Brahmin/Chettri	5
Dalit	9
Janajati	7
**Total**	**21**

We held four group interviews: two with women of reproductive age (*N* = 9, *N* = 7), and two with members of ‘health mothers groups’ (see background section above, *N* = 4, *N* = 2). We also conducted eight in-depth interviews with FCHVs in all three locations and 10 key informant interviews with District Health Officer, Public Health Officer, Public Health Nurse, FP/FCHV focal person of the district, three family planning providers, two Health Facility Operations and Management Committee members and the district co-ordinator for the intervention. Excerpts from these interviews are labelled ‘service provider’ in the text to preserve anonymity. The interviews with service providers were designed to answer specific evaluation questions rather than being central to the qualitative enquiry. They nevertheless contained some useful contextual details which we draw on here. Two fieldworkers observed FCHV work (including shadowing two FCHVs for two complete days) and made structured observations of health services (such as what commodities were available where) within the community.

Data for the evaluation as a whole took several forms: structured questionnaire, audio-recorded semi-structured interviews conducted in Nepali, field notes (e.g. structured observations of health facilities, observations about different activities, notes of informal conversations), and photographs (e.g. of health facilities, referral slips). We also analysed routine service data, comparing uptake of contraception before and after the intervention. Here we draw largely on the one-to-one in-depth and group interviews with women of reproductive age for our discussion as these were designed to explore contextual features of the intervention focusing on the women’s lives. Authors AA and SM conducted the interviews along with fieldworker, SK. The team developed interview guides based on prior guides developed by author CM for use in other settings, adapted for the local context. We asked women about what methods people used locally for contraception, and their fertility life histories, including their contraception and pregnancy histories. We asked them to tell us the stories of when they had used particular methods, why they used those methods, their relationship with their partner and how they had obtained the methods. We encouraged them to tell the stories in their own words, prompting them for details where needed. Details of the content of the interview guides are provided in the final evaluation report [15].

Each interviewer transcribed their own audio recordings (AA and SR interviews were sometimes transcribed by SK and SM). AA spot checked these for accuracy by comparing random sections of audio to the transcript to ensure the transcripts were a verbatim record. Interviewers added explanatory notes where needed (e.g. noting details such as when an interviewee indicated a part of her body by pointing). For interviews with the 15–49 year old women, we worked bilingually: AA worked with the Nepali transcripts and CM used English translations provided by bilingual staff (SM, SK) and professional translators. Where there were discrepancies or queries we used the original Nepali and authors AA, CM and SM worked together to improve the translation. All authors speak fluent English, and all but CM speak fluent Nepali. For the other interviews, we primarily used the English translations, returning to the Nepali transcripts to clarify complex passages.

AA and CM analysed the data using an iterative thematic approach [18] where we identified general themes, discussed which ones to explore in more detail, and refined those key themes through discussion and by reading and re-reading the transcripts to ensure we were appropriately representing the data.

The field team (SM and SK) were Nepali women in the same age range as the interviewees, which may have helped in recruitment and rapport although they were not from Myagdi. Observations of FCHV activities may have changed what was done, although it also provided opportunities to clarify activities and context. As with all qualitative interviews, data are co-created by interviewer and interviewee. We interpreted the narratives accordingly. For instance, women often told us they did not use contraception when their husband was away because it was unnecessary. We cannot know whether they ‘really’ did this, or whether they only said this to portray themselves as complying with community norms around extramarital sex. We treat such points as informative about community norms and of general circumstances and needs rather than as an indication of a ‘true’ account of what happened to a specific person. We base our interpretation on the various different types of data generated in this study and are informed by prior studies the HERD team has carried out in Nepal.

The study was approved by the Nepal Health Research Council (NHRC) and the Research Ethics Committee at the London School of Hygiene & Tropical Medicine (Reference 10345). All participants provided written informed consent to participate and we followed rigorous confidentiality, anonymity and data protection protocols, as required by our institutions.

## Results

While our evaluation did suggest that the training had improved the FCHVs’ interpersonal communication, crucially, the in-depth interviews highlighted how lack of information did not appear to be the major barrier to uptake of contraception compared with other sociocultural and health system barriers.

### Capturing nuance about the intervention context

The intervention was designed to help FCHVs provide better interpersonal communication on family planning, yet women’s accounts suggested that lack of information was not the main barrier they faced when they tried to obtain and use effective methods. When we asked our interviewees what types of methods people locally used to avoid pregnancy, they named effective methods (implants, injectables, OCPs and IUCDs) along with condoms, and many had used them. They knew where to obtain these methods from if they needed them. An existing programme, *Suaahara* [19], was operating in the area, and was mentioned by several interviewees as a source of information on nutrition and family planning *“There are discussions about having few children. Having more children causes trouble”* (IDI16).

There were supply-side problems. Our facility assessments [15] showed in most health posts only condoms, OCPs and injectables were available because of a lack of commodities and non-availability of trained providers of long acting reversible contraception (LARC). Some providers had been LARC-trained but lacked skills and confidence to implement this training. Implants, IUCD and medical abortion services were completely absent in 8 of the 15 VDCs we surveyed for the evaluation. In common with many parts of the country, permanent methods were only available in practice in the district via infrequent ‘sterilisation camps’. Only 13 health facilities in the entire district including the district hospital were licensed to provide medication abortion and were being supported by an international NGO to do so. Surgical abortions were only available at the district hospital.

Women in one of our group interviews complained that when they arrived at health facilities, the healthcare workers were often absent because they were running their own businesses such as medical stores elsewhere.

Even when contraceptives and providers were available, however, women often said they were turned away and told to come back on day 5 of their menstrual cycle:

*“I asked her [FCHV] to come with me [to the health post] saying that I wanted to get an implant and that I didn’t know anything about it. They gave me condoms telling me to come on the 5th day of menstruation.”* Later she reiterates *“It happens that I forget during menstruation and go on the 6*^*th*^*or 7*^*th*^*day. They tell us to come on the 5*^*th*^*day. I will go there without forgetting this [third] time.” (IDI13).*

Women reported being turned away for this reason even for methods where this was not necessary, provided she was reasonably certain she was not pregnant and a backup method was used for the first few days (e.g. injectables) [20]. One of the interviewees who had been turned away when she requested injectables because of the point in her cycle told us that she went on to conceive an unwanted pregnancy (IDI 16).

*“I had my menstruation when she [her first daughter] was 9 months old. I went to Darwang to get the injectable, but [PHC health worker] said that it has to cross a week of menstruation. Then I came back home. […]I don’t know what happened after I came back … I happened to conceive. […]When I talked about aborting it, no one in the family agreed. […]They told me not to abort saying that it will affect my body.” (IDI16)*

One service provider said that for such clients she tells them to use condoms and to come back on days 5 - 7 of menses. Some clients do not take the condoms, (e.g. the provider mentions they might say “If my husband used condoms I would not have had to come for Depo”). In this case, the provider reported saying to them:

*“If no baby is conceived, then that’s good but if you do get pregnant, then there are MA [Medical Abortion] services. You have to wait for your menstrual period in order to start the Depo. When your period starts, come after 5-7 days. If a baby is conceived, then come for MA.”* (service provider interview).

Many of the women we spoke to said they felt uncomfortable if the family planning provider was a man and would sometimes avoid the service for this reason:

“*It is a bit scary to talk with men [aboutfamilyplanning], we don’t even dare*.” And later in the same interview: “*As I said, it is ok to go to hospital if a woman [provider] is there. If a man is there then we can go to the FCHV-sister*.” (IDI17).

The qualitative interviews helped us obtain nuanced accounts of contraceptive choice and side effects. Women sometimes said they made their initial method choices based on friends’ and neighbours’ experiences —this was particularly given as the reason for using injectables. Others, however, said they would rarely discuss such things with others directly, with the possible exception of the FCHV “*I cannot say ‘sister what to do’ with others. FCHVs are [ …*] *doing the same work, so I do not feel embarrassed to talk to her*” (IDI17).

Women described worries such as IUCDs being painful or damaging the uterus, weight gain from injectables, or worries about physical weakness after sterilisation. While the prospect of side effects did seem to put some off -- *“a friend who used it [implant] became [fat] just like a rhinoceros. So I didn’t use it” (IDI7, who later says she is still thinking about trying an implant) –* crucially, based on what women told us, these worries did not necessarily translate into unwillingness to go ahead with using a particular method. Even direct personal experience of side effects did not necessarily cause women to discontinue use of reliable contraceptive methods. For instance, one interviewee said she suffered from dizziness and weight gain from using OCPs (IDI12) but continued using the method. She said she had been warned of possible side effects. One interviewee told us she had suffered from heavy, unpleasant, and disruptive bleeding for 6–7 months after a single dose of injectable. She visited a government health facility and was given pills to stop the bleeding. She then continued with OCPs as a method of contraception (IDI13). Interviewees commonly talked about experiencing disruptions to bleeds and/or heavy bleeding after using injectables. Some participants (IDI17,16,10) said they had carried out pregnancy tests after injectables stopped bleeds. One participant said she disliked injectables because of the irregular bleeds and dizziness she experienced. Despite this, she had not, at the time of interview, decided whether or not to discontinue use, largely because she was using it secretly and other options were unsuitable *“My husband does not know that I have had the injectable; he would shout at me if he knew.”* (IDI18). Injectables were also used secretly by others:

*Interviewer: Why [does your husband not talk about avoiding pregnancy]? Is it because he still has the desire to have more children?*

*“Well, maybe he has that desire. He neither knows that I have been using the injectable nor have I told him.” (IDI 16).*

Our attempts to take account of women’s reproductive lives in our analysis helped us understand the FCHV role. Our women interviewees and the FCHVs themselves told us that FCHVs actively gave advice about contraception (as opposed to limiting themselves to providing information and referrals). One interviewee told us her husband had wanted her to use OCPs or injectables and to obtain them from the hospital when she took her child for vaccination. She told him that she was worried about side effects of bleeding so he told her that the choice was up to her:

*“I could not decide on my own, and I went to [FCHV] and asked about what to do, how to do it. She said, “If you take medicine [OCPs] you might forget when walking here and there; you are a person with* children*. Use condoms for now and get the injectable when you go to the hospital.” (IDI17).*

In Myagdi, many husbands spend long periods working overseas. Their wives said they avoided using long-acting methods because they did not need them in their husbands’ absence, or because it would imply they were having extramarital sex. They also told us that their husbands sometimes returned from abroad with little or sometimes no warning, making it hard to plan contraception. One FCHV told us that a woman whose husband was arriving home that night from abroad had come to her for help because she did not have time to visit the health facility to ask for OCPs. The FCHV told us she gave her a single pill on the understanding that the woman would visit the health facility the next day (FCHV interview 8).

FCHV advice was not always complete or accurate – the FCHVs are not trained medical professionals – and poor advice sometimes had negative consequences. For instance, one of our interviewees said the FCHV advised her to stop using OCPs because of the risk (according to the FCHV) of long-term side effects such as heart or uterus problems, and advised her to switch to withdrawal instead. The FCHV even offered to phone the interviewee’s husband to tell him to comply. The woman followed the FCHV’s advice. She subsequently became pregnant, and blamed herself for the method failure:

*“We, husband and wife, discussed and used the withdrawal method as suggested by the FCHV. We were trying to throw [semen] outside [colloquial way to say withdrawal], but we threw it inside [Laughs].”* (*IDI 20).*

The woman already had two sons. She described her husband’s reaction to her pregnancy:

“*At first [ …]he said what happened was good [Laughs]. He wanted to have a daughter and said that his wish had come true. Then I yelled at him, after which he asked me to calm down and do what I want.” (IDI 20).*

She said that the FCHV had told her where to go and she opted for medication abortion from a nearby government health post. The pregnancy ended without any complications. It is unclear whether the FCHV had taught interviewee 20 about withdrawal, but few of our interviewees said they were familiar with the method:

*Interviewer: “Have you* heard *of throwing it outside after having relationship? Has your husband ever done anything like that?”*

*“Why would a man agree to do that [laughing]? Either we have to get the injection or take pills” (IDI13).*

In another location, one of the FCHVs -- who had received the training we were evaluating -- told us she provided information about withdrawal to women, telling them that if they are using withdrawal they do not need to use any other methods. She also said she provided information to women complaining about side effects of other methods: she said she tells them that days 14–18 of a menstrual cycle constitute the “unsafe period” and if they abstain from sex during this period then they will not have to use another method. She also said that she tells women to start oral contraceptive pills within 4–5 days of menstruation, and inaccurately informs them that pills will not work if started after the seventh day (they will work, just not immediately) [20]. IPC training may have helped FCHVs to serve their communities better, but information accuracy is clearly still a problem. Whether the FCHV training had improved the situation from the client perspective was not clear; however, FCHV clients took referral slips to providers which suggests that they were acting on discussions they had had with FCHVs.

### Women’s privacy and information sharing

Women frequently mentioned privacy concerns relating to family planning services. This was particularly pronounced for abortion services – for instance, one woman told us that in order to stay anonymous, she travelled to Pokhara (i.e. out of district) to obtain surgical abortion. Yet healthcare staff may treat FCHVs as though they are part of the health system and share information about clients with the FCHV and vice versa, so she may be party to the most intimate aspects of women’s lives even if they have not told her about their health facility visits. One woman told us her relative paid to attend a private facility so that the community would not find out about her injectable contraceptive use, and as mentioned earlier our interviewees talked about using injectables secretly.

Nevertheless, one service provider told us how they ask the FCHVs to follow up family planning clients and if the client discontinues method use, they ask FCHVs to find out why.

*“We [health workers] call FCHVs to follow up people who are using the service. We also ask them to observe those people and if they have any problem call us for help. Or we ask the client to meet the nearest FCHV if they have any problems. Nowadays, we keep in contact with clients with phone calls because network coverage has increased.*

*Interviewer: Do they call you?*

*“Yes, they do. If they become irregular [*i.e. *if they don’t come for family planning services at the time they should come] then we enquire through the FCHV, and then we contact them.” (Service provider interview).*

Women’s worries about information leakage may be well founded: FCHVs seemed to be expected to know women’s reproductive status in their community. They were routinely praised for their knowledge by incoming NGOs and any programme where they are needed to access members of the community. When their knowledge was not complete, they said they were sometimes scolded by providers. While FCHVs may be accepted sources of information on family planning, this does not necessarily translate into women trusting them with their personal information where concerns around maintaining confidentiality are more likely to arise.

### Better understanding of process

Our interviews ensured we had a better understanding of how the intervention was being carried out in practice. We interviewed a number of women who said they had attended ‘health mothers groups’ where they had discussed nutrition and family planning, including being shown real examples or photographs of devices such as implants, IUCDs, OCPs and injectables (IDI 5,7,10,11,13,14), and we also observed these happening in practice. Based on the women’s and the FCHV’s accounts, equally or more important for family planning was the FCHV’s role in supplying or advising on methods of contraception or abortion on a one-to-one basis. FCHVs were trusted in communities and played an important bridging role to health services, via referrals and working with providers. Our observations and discussions with women and FCHVs in the communities indicate that women visit FCHVs and ask for advice, FCHVs visit households and, in some cases, phone women they cannot reach easily in person, to provide information and referrals.

*“Everybody has faith in the FCHV [ …*] *She has taken responsibility, she is the one who is knowledgeable, she* has *understood too. So in my view it is better to receive the information [about family planning] from her [rather than other women].” (IDI17)*

While the FCHVs were generally enthusiastic about the training they received during the intervention, it was not always clear what impact it had had on their practice. For instance, one of the FCHVs said the training had helped her communicate better, though quickly qualified this:

*“Before we used to talk impolitely or directly but now we have learned ways of being friendly with the woman so that we can get her inner thoughts [ …*] *It has made it easier to make people understand. To share the inner feelings …****before also it was not so difficult****for me to deal and speak with the women.” (FCHV 2, emphasis added).*

One unexpected finding that emerged because of our qualitative data collection was to do with unintended effects of our evaluation methods. In an attempt to track referrals for the evaluation, we had asked FCHVs to provide women with referral slips – pre-printed pieces of paper on which the FCHV wrote what contraceptive method(s) the woman was interested in. The FCHV asked the women to give these to the providers. Some of the women told us that the slips had helped them by allowing them to express what method they were interested in without having to say it out loud.

*Interviewer: “Now you will go to the health post to get injectable [Depo]. Would it be difficult for you after you go to the health post if you did not have that card?”*

*“It would obviously be difficult. [Laughing]*

*[…]*

*It would be difficult to speak. As I said I cannot directly say, ‘I want to get injectable [Depo] to prevent pregnancy.’ It is difficult for everyone to say directly.” (IDI17)*

Interviewee 18 said she had been turned away by the health provider and told to discuss injectable use with her husband. Having a written document from the FCHV seemed to have helped her gain access to the method.

*“They did not give me [the injectable], and I went back [to the FCHV] and said that this happened; they did not give it to me. And she gave me a card saying, ‘If that’s the case, I will write a card. They will give it to you after you take* this*.’*

*[…].*

*She [health worker] gave it to me after I took that card there [laughs]”*

It was unclear from the interviews whether all the slips that women told us about were the ones we had distributed to FCHVs for evaluation purposes. Regardless, it was clear that referral slips were not neutral, and rather than only tracking referrals, as intended, they may have directly influenced contraceptive access and therefore possibly also uptake.

## Discussion

Women in our study did not seem to be avoiding contraception because of being ill-informed, rather they were often being prevented from accessing it, suggesting first, that the intervention we were evaluating was addressing only a small part of the problem and second, that the implicit model underpinning the intervention appeared to be very limited given that information and communication from FCHVs, while important in general, did not seem to be the key limiting factor requiring intervention to improve contraceptive uptake. Service data were limited but provided no particular evidence the intervention had increased contraceptive uptake. Incorporating the qualitative enquiry into the lives of women who would be the ‘targets’ of FCHV information as well as interviewing the FCHVs themselves provided contextual data that helped us identify ways in which the intervention could be understood and improved, even in the absence of a large ‘effectiveness’ type study which was not possible and probably not desirable given the limitations that we identified at the start.

We uncovered information that could be helpful for adapting services so that they are more tailored to the local context. For instance, the fact that women whose husbands were away for long periods of time did not wish to use LARCs and were sometimes unprepared when husbands returned without warning. These women, along with the women whose partners did not withdraw in time, could potentially benefit from emergency contraceptive pills (ECPs) yet these were hardly mentioned in the interviews. Feasibility and acceptability of promoting ECPs could be investigated, perhaps even including distributing ECPs via FCHV networks to reduce travel time to access the method.

Privacy appears to be an issue that needs addressing. It is unclear to what extent information sharing between health facilities and FCHVs is expected, but our experience of meetings discussing reproductive health interventions and our observations in the field suggest that it is taken for granted that FCHVs and health facilities would share information. While some women may be happy for this to happen, for others it could be problematic. For instance, one proposal we witnessed at a Kathmandu-based meeting, was to encourage health facilities to inform FCHVs about women who were due for a new dose of injectables so that the FCHV could go to visit the woman to remind her. However, for secret injectable users, a home visit of this sort could be harmful. As far as we know these visits were not carried out (objections were raised at the time) but the fact it was considered at all suggests that there is an assumption that information sharing would be acceptable (or that its benefits outweigh its costs), and underscores the importance of ensuring community members are involved in discussions about interventions that affect them, not only to ensure that their needs are met, but also to help avoid unintended harms.

Our evaluation methods unexpectedly became an intervention, in that they directly affected the pathway between FCHV communications and contraceptive method uptake by mitigating women’s embarrassment at asking for methods. This unintended effect appears to have been positive – and might be worth looking into developing as a way to help women access methods more comfortably. However, this highlights the need to check for unintended consequences not only of unintended harms of interventions [21] but also evaluations. A qualitative component that goes beyond ‘process evaluation’ to explore the context of the intervention from the point of view of the intended beneficiaries and other stakeholders has the potential to help with this.

Our findings challenge the persistent ‘deficit’ model of family planning interventions where women living in poverty are assumed to be ill-informed and resistant to using effective contraception, simply needing more information, or be disabused of myths, in order to increase contraceptive uptake. Our interviewees described enduring severe side effects of contraception and going to great lengths to obtain effective methods in order to avoid pregnancy. Surveys often ask women who discontinued contraceptives why they did so and collect data on side effects in this context. However, we lack estimates of the prevalence of women who continue with methods while enduring unpleasant side effects because relevant questions are generally omitted. Our findings suggest analysis of survey data affected by such omissions could lead to misunderstandings of contraceptive motivation and practice e.g. experiencing side effects being construed as evidence that side effects ‘cause’ switching or discontinuation of methods because only women who discontinue or switch methods are asked about them, when the reality is likely to be more complex.

Our evaluation took place over a comparatively short period of time and the in-depth interviews could not cover every area that we would have liked to explore. Despite these limitations, the approach yielded several interesting areas for further exploration and action; a wider enquiry would presumably yield even more.

FCHVs play a key role for family planning in Nepal: there are few health facilities in remote areas and so women living long distances away have limited access to other options. Nepal has made commitments to improved family planning as part of FP2020, as well as commitment to “leaving no one behind” and “reaching the unreached” in family planning [22]. FCHVs can play a crucial role for this, but need support. Our findings illustrate FCHVs were taking on responsibilities that would better be held by the formal health system such as providing family planning advice (rather than simply promoting family planning as they are intended to do). There is an opportunity with the implementation of federalism in the health sector to revisit community health systems including the role of FCHVs. A new cadre of paid, trained community health workers could help to meet some of the need for family planning services outreach and support FCHVs. The skills mix of any new staff should match local context and needs. FCHVs could be further supported by revising FCHV training manuals and tools within the routine health system, and with supportive supervision from health workers to address information gaps and misconceptions.

FCHVs have unparalleled access to communities across Nepal and their bridging role to services is already long established. They have the potential to help transform health systems’ engagement with the communities they serve. Community engagement is a key action area within the Global Strategy for Maternal, Newborn, Child and Adolescent health [23] and a cornerstone of rights-based healthcare. Community members may have excellent ideas about how to use limited resources to improve care as is the case in other settings [24, 25]; at present there is little opportunity to put this to the test. While FCHVs are already in a position to bring views of their neighbours to health providers, we found little evidence that they or healthcare providers saw this as a major part of their role. This is an area that could profitably be developed. For instance, a participatory approach could help find solutions to the issues of privacy we identified. Clients could, and should, be involved in dialogue about how their information is shared, and about how this should be managed to help ensure that individuals understand what any sharing between health providers and FCHVs might mean for them and others (for instance, they might wish to consent to such sharing, or be able to opt out of sharing). Involving clients, FCHVs and family planning providers in a dialogue together to plan programmes will also help ensure that possible unintended consequences of any actions are considered by those directly affected.

## Conclusion

Including in-depth interviews exploring women’s experiences of fertility and contraception within our evaluation helped contexualise the intervention and yielded nuanced data on contraceptive choices, needs, and areas for future action. The in-depth qualitative component also – unexpectedly – drew our attention to unanticipated effects of our own evaluation strategies – the effects of the introduction of referral slips on contraception access – that would otherwise have gone unnoticed.

Attending to women’s lives and experiences allowed us to begin to understand the likely pathways from intervention to effect from the user point of view without narrowly focusing only on the intervention, helping us identify possible areas for action that might otherwise have been missed. By increasing attention to context of family planning interventions, and the crucial role of understanding people’s experiences within those contexts, we can build a body of evidence that can not only inform future interventions within that context, but also build better knowledge of contextual factors likely to be important elsewhere.

## Data Availability

The datasets generated and/or analysed during the current study are not publicly available as they contain sensitive information and data could potentially identify participants.

## Abbreviations

ECP: Emergency contraceptive pill
FCHV: Female Community Health Volunteers
IUCD: Intrauterine contraceptive devices
NGO: Non-governmental Organisation
OCP: Oral contraceptive pill
VDC: Village Development Committee
